# Hepatoprotective activity of *Eugenia jambolana* Lam. in carbon tetrachloride treated rats

**DOI:** 10.4103/0253-7613.48888

**Published:** 2009-02

**Authors:** S.S. Sisodia, M. Bhatnagar

**Affiliations:** Department of Pharmacology, Bhupal Nobles' Girls College of Pharmacy, Udaipur, Rajasthan, India; 1 Laboratory of Animal Cell Biotechnology, Department of Biotechnology, University College of Science, M. L. S. University, Udaipur, Rajasthan, India

**Keywords:** Carbon tetrachloride, *Eugenia jambolana* Lam., marker enzymes, methanolic extract

## Abstract

**Objective:**

To estimate the hepatoprotective effects of the methanolic seed extract of *Eugenia jambolana* Lam. (Myrtaceae), in Wistar albino rats treated with carbon tetrachloride (CCl_4_).

**Materials and Methods:**

Liver damage in rats treated with CCl_4_ (1ml/kg/Bw, administered subcutaneously, on alternate days for one week) was studied by assessing parameters such as serum glutamate oxaloacetate transaminase (SGOT), serum glutamate pyruvate transaminase (SGPT), alkaline phosphatase (ALP), acid phosphatase (ACP) and bilirubin (total and direct). The effect of co-administration of *Eugenia jambolana* Lam. (doses 100, 200 and 400 mg/kg p. o.) on the above parameters was investigated. These biochemical observations were supplemented by weight and histological examination of liver sections. Liv.52^®^ was used as positive control. Data were analyzed by one way ANOVA, followed by Scheff's/Dunnett's test.

**Results:**

Administration of *Eugenia jambolana* Lam. (doses 100, 200 and 400 mg/kg p. o.) significantly prevented carbon tetrachloride induced elevation of serum SGOT, SGPT, ALP, ACP and bilirubin (total and direct) level. Histological examination of the liver section revealed hepatic regeneration, after administration of various doses of *Eugenia jambolana* Lam. The results were comparable to that of Liv.52^®^.

**Conclusion:**

The study suggests preventive action of *Eugenia jambolana* Lam. in carbon tetrachloride induced liver toxicity. Hepatic cell regeneration process was dose dependent.

## Introduction

The liver, in vertebrate body, performs many vital functions, including metabolic and detoxification activities. A number of chemical agents and routine drugs produce cellular as well as metabolic liver injury. Therefore, many herbal and other indigenous sources have been adequately explored for the safe and effective hepatoprotective action. *Eugenia jambolana* Lam. (Myrtaceae), popularly known as *Jamun*, is being widely used to treat liver dysfunctions and diabetes by the traditional practitioners for over many centuries.[1–3] The bark of this plant is astringent, antihelmenthic, antipyretic, antidysentric and useful in certain urinary disorders, excessive thirst, hemorrhages, ulcer and vaginal discharges.[4] The juice is helpful in treating inflammation and swelling on the liver and spleen.[5] The chemical constituents of the seed of *Eugenia jambolana* Lam. are gallic acid, ellagic acid, corilagin, ellagitannins, isoquercetin, quercetin, caffeic acid, ferulic acid, guaiacol, resorcinaldimethyl ether, lignaglucoside, veratrole, β - sitosterol, palmitic acid etc.[1,5]

In view of the above, the present study was carried out with the aim of evaluating the hepatoprotective properties of the seed extract of *Eugenia jambolana* Lam., in experimental rat model of liver injury induced by carbon tetrachloride.

## Materials and Methods

### Plant material and chemicals

The seeds of the plant *Eugenia jambolana* Lam. was collected from a rural area near Udaipur (Rajasthan) city and was purified using the absorption method (by keeping them in contact with brick powder). After purification, the fine powder of the seeds was packed in high quality filter paper and the successive solvent extract (methanol) was prepared by continuous extraction method, with the help of a soxhlet extractor. After vacuo evaporation, the crude extract was suspended in 0.5% carboxy methyl cellulose (CMC) and stored in a refrigerator for further use. Liv.52^®^ (The Himalaya Drug Company, Bangalore, Batch No. 41001CL, Mfg. Date: October 2004, Exp. Date November 2007) syrup was used as positive control. Carbon tetrachloride (Ranbaxy Laboratory Ltd., Batch No. 6FMV0468R) was used to induce hepetotoxicity.

### Experimental Animals

Male albino rats (Wistar strain) weighing 200–225g of either sex were used for the present study. The animals were housed in polypropylene cages at controlled temperature (26 ± 2°C), relative humidity (60 ± 5%) and light conditions(12 -12 hours day night cycle). The rats were fed with standard laboratory diet and drinking water was given through a drinking bottle, throughout the experiment. The animals were maintained as per the CPCSEA regulations and the study was approved by the IAEC at Bhupal Nobles' College of Pharmacy, Udaipur (Rajasthan).

### Experimental induction of hepatotoxicity

Liver toxicity was induced in rats by administrating carbon tetrachloride (CCl_4_) subcutaneously (sc), in a suspension of liquid paraffin (LP; 1: 2 v/v) at a dose of 1 ml/kg body weight, on alternate days, for one week.[6]

### Experimental design

The rats were divided into six groups I – VI, each group consisting of six rats. The rats in group I served as control and received subcutaneous administration of liquid paraffin only 1 ml/kg on alternate days, for one week. Group II rats were given carbon tetrachloride (CCl_4_) sc, in a suspension of liquid paraffin (1 : 2 v/v), at a dose of 1 ml/kg body weight, on alternate days, for a week. Group III rats were given Liv.52^®^ orally daily and carbon tetrachloride (CCl_4_), in a suspension of liquid paraffin (1 : 2 v/v), at a dose of 1 ml/kg body weight, on alternate days, for a week, subcutaneously. Group IV – VI were orally administered the extract of *Eugenia jambolana* Lam. (100, 200 and 400 mg/kg) respectively daily and carbon tetrachloride (CCl_4_), in a suspension of liquid paraffin (1 : 2 v/v) at a dose of 1 ml/kg body weight, on alternate days, for a week, by the subcutaneous route. The daily food consumption was monitored. The different doses of *Eugenia jambolana* Lam., LP and CCl_4_ were administered to the rats daily, between 8.00 a.m. and10.00 a.m.

On the eighth day, the animals were sacrificed by decapitation. Through an incision made on the jugular vein, blood was collected. The blood and serum were separated by centrifugation and used for estimation of biochemical parameters, that is glutamate oxaloacetate transaminase (GOT),[7] glutamate pyruvate transaminase (GPT),[7] alkaline phosphatase (ALP),[8] acid phosphatase (ACP) and bilirubin (total and direct).[8]

### Statistical analysis

The results of the biochemical estimations are reported as mean ± SD of six animals in each group. The data were subjected to one-way analysis of variance (ANOVA). This was followed by Scheff's/Dunnett's test, to determine the statistical significance of the difference in enzyme activity and other parameters. The level of significance was *P*<0.05.

### Histology

The liver tissue was excised from the animals, washed with the normal saline to remove blood, fixed in 10% buffered neutral formalin for 12 hours and processed for paraffin embedding. Sections of 5*μ*m thickness were cut using a rotary microtome. The sections were processed and passed through graded alcohol series, stained with alum haematoxylin and eosin,[9] cleared in xylene and cover slipped in DPX. Histological examination was done under AO Star microscope.

## Results

### Serum marker enzymes

The levels of marker enzymes, viz. GOT, GPT, ALP, ACP and Bilirubin (total and direct) were significantly increased in group II carbon tetrachloride treated animals, as compared to the control untreated group I. The groups III – VI, treated with Liv.52^®^ and different doses of *Eugenia jambolana* Lam., followed by carbontetrachloride, showed significant decrease in the level of serum marker enzymes, as compared with the carbon tetrachloride treated group II [Tables 1 and 2].

**Table 1:** The effect of methanolic seed extract of *Eugenia jambolana* Lam. on CCl_4_ induced hepatotoxicity in rats

*Group/Treatment*		*GOT U/L*	*GPT U/L*		*ALP K. A. Units*	*ACP K. A. Units*		
Group I		37.78 ± 7.26	46.69 ± 10.74		12.45 ± 2.99	6.51 ± 0.52
Control with LP						
Group II		93.16 7.92+++	99.68 ± 11.10+++		58.84 ± 14.75+++	14.01 ± 2.48+++
LP + CCl_4_						
Group III		52.14 ± 3.14***	55.95 ± 5.57***		27.79 ± 3.99***	9.33 ± 0.98**
LP + CCl_4_ + Liv.52^®^ (1 ml/kg)						
Group IV		75.11 ± 3.57***	78.66 ± 4.87**		54.71 ± 3.49	11.92 ± 0.49
LP + CCl_4_ + MT (100 mg/kg)						
Group V		51.50 ± 2.08***	73.94 ± 3.82***		43.61 ± 5.54*	10.71 ± 0.80*
LP + CCl_4_ + MT (200 mg/kg)						
Group VI		53.28 ± 6.97***	68.50 ± 4.11***		40.46 ± 3.05*	10.72 ± 1.03*
LP + CCl + MT (400 mg/kg)				

One Way F	64.71	41	34.41	24.41		
df	5, 30	5, 30	5, 30	5, 30		
ANOVA*P*	<0.001	<0.001	<0.001	<0.001		

Values are represented as mean ± SEM (n = 6) in each group.

+++ *P* ≤ 0.001 as compared to control rats.

* *P* ≤ 0.05

** *P* ≤ 0.01

*** *P* ≤ 0.001 as compared to CCl_4_ treated rats. LP- Liquid parafin

**Table 2:** The effect of methanolic seed extract of *Eugenia jambolana* Lam. on bilirubin in CCl_4_ induced hepatotoxicity

*Group/Treatment*			*Bilirubin (mg/dl)*	
	
			*Total*	*Direct*
Group I			0.74 ± 0.04	0.27 ± 0.03
Control with LP				
Group II			0.83 ± 0.02+++	0.41 ± 0.33+++
LP + CCl_4_				
Group III			0.75 0.01***	0.31 ± 0.02***
LP + CCl_4_ + Liv.52^®^ (1 ml/kg)				
Group IV			0.81 ± 0.03	0.31 ± 0.03***
LP + CCl_4_ + MT (100 mg/kg)				
Group V			0.79 ± 0.01**	0.34 ± 0.03**
LP + CCl_4_ + MT (200 mg/kg)				
Group VI			0.75 ± 0.02***	0.32 ± 0.03***
LP + CCl_4_ + MT (400 mg/kg)				

One Way F	12.822	15.06		
ANOVA df	5, 30	5, 30		
*P*	< 0.001	< 0.001		

Values are represented as mean ± SEM (n = 6) in each group.

+++ *P*≤ 0.001 as compared to control rats.

*P≤ 0.05

** *P*≤ 0.01

*** *P*≤0.001 as compared to CCl_4_ treated rats.

### Histological examination

**Figure 1:**
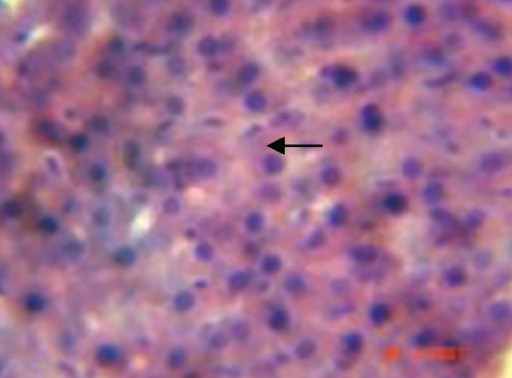
Group I (Control): Section of liver with normal cell structure (→ arrow). 40×

**Figure 2:**
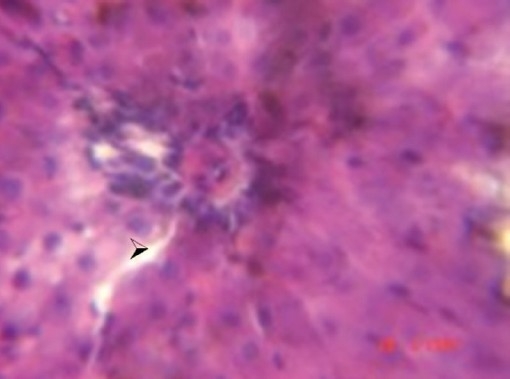
Group II (CCl_4_) Section of liver showing centriolobular necrosis (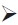; arrow). 40×

**Figure 3:**
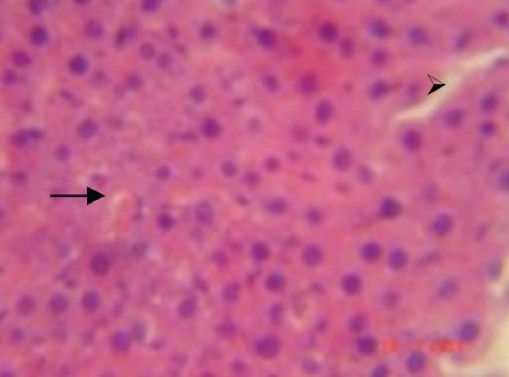
Group III (Liv.52^®^): Section of liver showing reduced necrotic area, normal cell structure (→ arrow) and necrotic cell(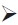; arrow).40×

**Figure 4:**
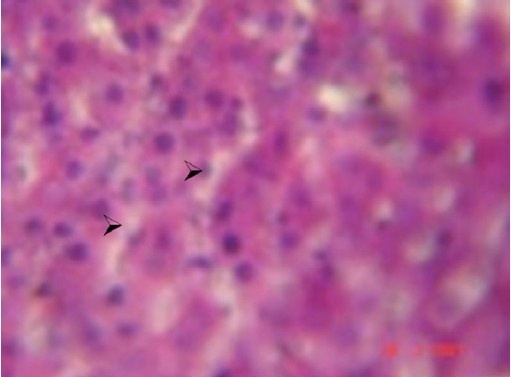
Group IV (EJ 100): Section of liver showing reduced necrotic area. Note the vacuolar degeneration (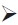; arrow). 40×

**Figure 5:**
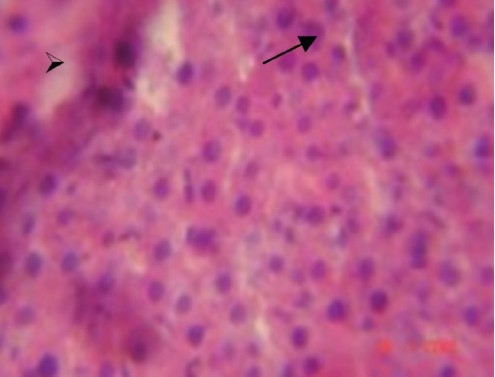
Group V (EJ 200): Section of liver showing comparatively lesser area of necrosis and vacuolar degeneration, normal cell structure (→; arrow) and necrotic cell (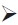; arrow) (40×)

**Figure 6:**
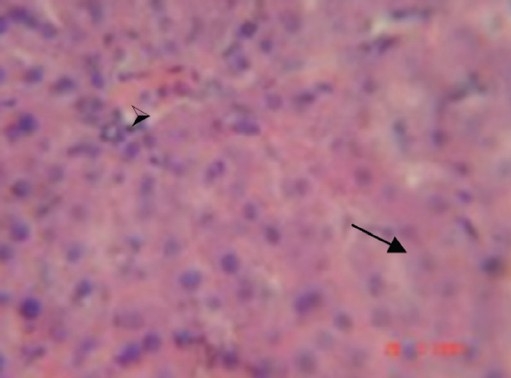
Group VI (EJ 400): Section of liver showing significantly reduced necrotic area (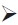; arrow) and vacuolar degeneration, normal cell structure (→; arrow) (40×)

During the histological examination of liver sections of the control group [Figure 1], it was observed that the central vein was prominent, with normal hepatocytes. In the carbon tetrachloride intoxicated group [Figure 2], centriolobular necrosis was observed. In the histological profile of the Liv.52^®^ treated group [Figure 3] and the different groups treated with *Eugenia jambolana* Lam [Figures 4–6], there was less centriolobular necrosis and hepatocytes showing regeneration activity.

## Discussion

Carbon tetrachloride, a widely used experimental hepatotoxicant, is biotransformed by cytochrome P - 450 systems to produce the trichloromethyl free radical (CCl_3_•) that causes lipid peroxidation and, thereby, produce liver damage.[10–12] Carbon tetrachloride produces the dose dependent hepatotoxicity by directly affecting the liver, causing lipid peroxidation.[11] The mechanism of the action of carbon tetrachloride is complex, multifactorial and not completely understood. When administered, carbon tetrachloride accumulates in hepatic parenchymal cells, which is metabolized to free radical CCl_3_•. The free radicals react with molecular oxygen to produce peroxy radicals (H_2_O_2_, O_2_ and •OH due to incomplete reduction of molecular oxygen), thereby causing oxidative destruction of polyunsaturated fatty acids.[13] These activated radicals bind covalently to the macromolecules and induce peroxidative degradation of membrane lipids of endoplasmic reticulum, rich in polyunsaturated fatty acids.

Lipid peroxidative degradation of biomembrance is one of the principle causes of hepatotoxicity.[14] In acute hepatic necrosis, increase in the serum level of glutamate pyruvate transaminase (GPT) is followed by an increase in the level of glutamate dehydrogenase (GDH), which is indicative of liver mitochondrial injury.[15] This is evidenced by an elevation of the serum marker enzymes GOT, GPT, ALP and ACP in the carbon tetrachloride treated rats.[16,17] When liver cell plasma membrane is damaged, a variety of enzymes, normally located in the cytosol, are released into the blood and their estimation is a useful quantitative marker of the extent and type of hepatic cell damage.[18] In the present investigation, treatment with different dosages of the extract of *Eugenia jambolana* Lam. (100, 200 and 400 mg/kg p. o.) significantly reversed these elevated marker enzymes, viz. - GOT, GPT, ALP, ACP and bilirubin (total and direct), and the results obtained were comparable with those of the Liv.52^®^ treated group.

A unique feature of the liver tissue is its ability to regenerate. The cell repair mechanisms are influenced by phospholipids coupled with a rise in thymilidate synthetase and thymidine kinase level in the liver, reaching a peak at 72 hours, indicating liver regeneration.[19] Hepatic cell damage and recovery proceeded simultaneously. However, it is inhibited by repeated dosage of carbontetrachloride.[20]

In our study, 400mg/kg p. o. dose was highly effective, as compared to other dosages. It is thus concluded that the methanolic extract of *Eugenia jambolana* Lam., at an oral dose 400mg/kg/day, is effective against the hepatotoxicity caused by carbon tetrachloride. In our study, a comparative histopathological study of the liver from different groups, also shows the hepatoprotective efficacy of *Eugenia jambolana* Lam.

Further profound studies are required to establish the therapeutic potential and safety of the drugs of herbal origin, in the treatment of hepatotoxicity.

In conclusion, our study demonstrates that the seed extract of *Eugenia jambolana* Lam. can be effective treatment against liver injury.
